# Using electronic technology to improve clinical care – results from a before-after cluster trial to evaluate assessment and classification of sick children according to Integrated Management of Childhood Illness (IMCI) protocol in Tanzania

**DOI:** 10.1186/1472-6947-13-95

**Published:** 2013-08-27

**Authors:** Marc Mitchell, Bethany L Hedt-Gauthier, Daniel Msellemu, Melania Nkaka, Neal Lesh

**Affiliations:** 1 Department of Global Health and Population, Harvard School of Public Health, 665 Huntington Avenue, Boston, MA 02115, USA; 2 D-Tree International, Weston, MA, USA; 3 Department of Global Health and Social Medicine, Harvard Medical School, Boston, MA, USA; 4 Ifakara Health Institute, Dar es Salaam, Tanzania; 5 D-Tree International, Dar Es Salaam, Tanzania; 6 Dimagi Inc, Cambridge, MA, USA

## Abstract

**Background:**

Poor adherence to the Integrated Management of Childhood Illness (IMCI) protocol reduces the potential impact on under-five morbidity and mortality. Electronic technology could improve adherence; however there are few studies demonstrating the benefits of such technology in a resource-poor settings. This study estimates the impact of electronic technology on adherence to the IMCI protocols as compared to the current paper-based protocols in Tanzania.

**Methods:**

In four districts in Tanzania, 18 clinics were randomly selected for inclusion. At each site, observers documented critical parts of the clinical assessment of children aged 2 months to 5 years. The first set of observations occurred during examination of children using paper-based IMCI (pIMCI) and the next set of observations occurred during examination using the electronic IMCI (eIMCI). Children were re-examined by an IMCI expert and the diagnoses were compared. A total of 1221 children (671 paper, 550 electronic) were observed.

**Results:**

For all ten critical IMCI items included in both systems, adherence to the protocol was greater for eIMCI than for pIMCI. The proportion assessed under pIMCI ranged from 61% to 98% compared to 92% to 100% under eIMCI (p < 0.05 for each of the ten assessment items).

**Conclusions:**

Use of electronic systems improved the completeness of assessment of children with acute illness in Tanzania. With the before-after nature of the design, potential for temporal confounding is the primary limitation. However, the data collection for both phases occurred over a short period (one month) and so temporal confounding was expected to be minimal. The results suggest that the use of electronic IMCI protocols can improve the completeness and consistency of clinical assessments and future studies will examine the long-term health and health systems impact of eIMCI.

## Background

There is considerable interest in the use of mobile technology to improve health care in low and middle income countries. However, there has been a relative shortage of evidence demonstrating its impact on health outcomes [1-3]. This study explored whether mobile technology can improve the quality of care in the area of child health and in particular in the delivery of the widely used Integrated Management of Childhood Illness (IMCI) protocol for classifying and treating common causes of death including pneumonia, diarrhoea, malaria, measles, and malnutrition. A multi-country evaluation coordinated by the WHO concluded that IMCI has the potential to improve quality of care, reduce the cost of treatment, and reduce under-5 mortality when used correctly [4-7]. However, despite a worldwide effort, the use and impact of IMCI protocols remains limited due to the expense of training, the lack of sufficient supportive supervision, the tendency for health workers to follow protocols less rigorously over time, and insufficient resource and policy support [8,9].

When facing sub-par performance, most countries and most health systems still rely on training and supervision as the basis for quality management. Unfortunately, due to the uneven quality of training and general lack of supervision, this approach is often not sufficient [10]. The emerging use of mobile technology in health offers new strategies for improving quality of health care [3,11,12]. The availability of mobile phones has grown significantly in low-income countries. In 2010 over 45% of households owned at least one mobile phone in Tanzania [13] and a recent unpublished study by the telecom companies in Tanzania now puts that estimate at 70%. This suggests that the use of cell phones for improving care in low-income countries is both feasible and affordable.

A preliminary study suggested that some of the barriers to correct use of IMCI can indeed be alleviated with the use of mobile technology [14]. This paper evaluates the impact of mobile technologies on the quality of IMCI implementation at multiple health facilities in Tanzania, including completeness of assessment, accuracy of classification, and consistency of care across facilities.

## Methods

### Setting

Tanzania adopted IMCI protocols as national policy in 1998 [15]. The study was conducted between May 2008 and December 2009 in four districts in Pwani Region, two semi-urban (Bagamoyo and Morogoro Urban) and two rural (Mkuranga and Morogoro Rural). The study was restricted to primary care facilities where children are seen primarily by clinical officers. Throughout Tanzania, these facilities typically provide the front line of care to children under-five and provide referral for more serious cases for care at hospitals. Clinical officers have three years of post-secondary school training and are registered as medical practitioners by the Ministry of Health.

### Clinic recruitment

All primary care facilities with at least one health worker trained in IMCI were eligible for the study. Eighteen facilities were randomly selected from the 227 facilities that had sufficient volume of patients (minimum expected five per day) across the four districts and the one health worker trained in IMCI at the facility was invited to participate in the study. If there was more than one health worker trained in IMCI, then a single health worker was randomly selected and invited to participate.

### Intervention

**Figure 1:**
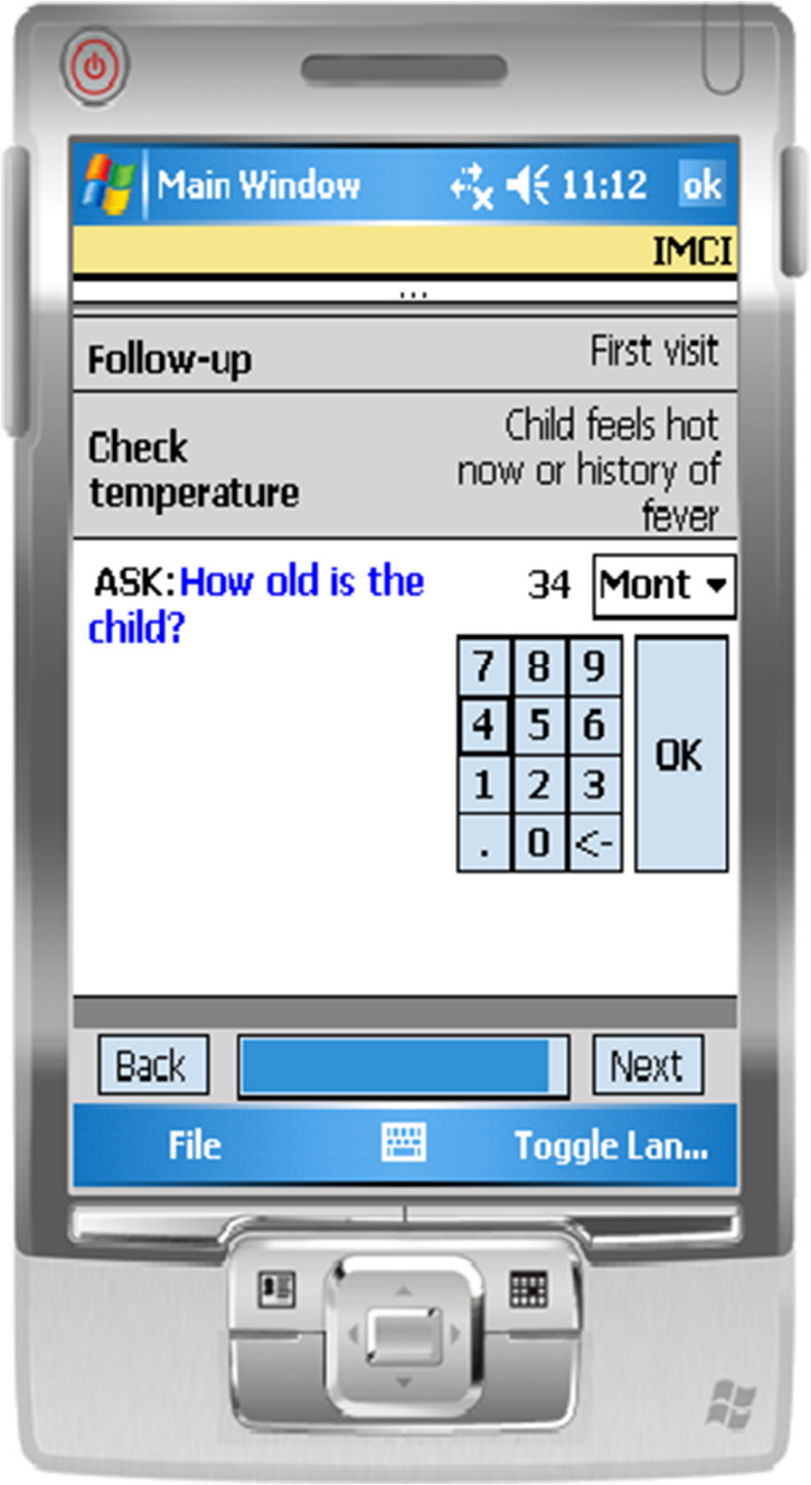
Screenshot of eIMCI.

In Tanzania, health workers trained in IMCI receive a paper booklet to help facilitate following of IMCI protocols [16]. We developed an electronic version of IMCI (eIMCI) to run on a Personal Digital Assistant (PDA) (Figure 1). This study used PDAs for implementation; however eIMCI could be easily adapted and implemented on other electronic mediums including laptops, tablets, or smartphones.

The eIMCI protocol followed the same protocol in the current paper system guiding healthcare workers step-by-step through the child’s assessment, classification, treatment, and communication of instructions to the caregiver. During the assessment phase, results were recorded by the healthcare worker directly into the PDA. For symptoms that required additional follow-up to assess severity, the algorithm prompted the necessary questions as per the IMCI protocol. Based on the assessment, the eIMCI tool provided classification, treatment and communication recommendations in accordance to the IMCI protocol. For steps that did not require data gathering, for example the communication of an instruction to a mother, the healthcare worker selected the “next” button in the PDA to indicate that the step was completed. All eIMCI instructions were available in English and Swahili.

### Implementation and data collection

At each site, data collection occurred over two phases – the first phase under the current paper based system (pIMCI) and the second phase under eIMCI. Between the two phases, the health worker was introduced to eIMCI and given instructions on how to operate the tool. No additional training on IMCI was given. Both the paper phase and the eIMCI phase of observations occurred over three to six clinic days at each site.

Any child age 2–59 months visiting the facility for their first sick child visit was eligible for inclusion. If the child’s caregiver consented to participate, an external data collector silently observed the clinic visit. Occurrence of critical pieces of the assessment and classification, treatment and recommended follow-up instructions were documented in a study form. The data collectors had university degrees, were experienced data collectors at a research organization in Tanzania and received extensive training to minimize observer and desirability bias. Following the standard clinic visit, the child then proceeded to an IMCI expert, individuals who lead national-level IMCI trainings and were available at the clinic as part of the study. The expert completed a second full assessment of the child and provided their classification, suggested treatment and follow-up instructions for the child. During the study, the classification, treatment and advice of the expert was the basis of final treatment of the child.

### Primary and secondary outcomes

The primary outcome for this study was completed assessment on each of the 15 critical items of the IMCI encounter (assessment items listed in Tables 1 and 2). These items match those used in previous studies that looked at the quality of IMCI [7]. Ten of the 15 assessment items were included in the eIMCI system. Five of the items that were not necessary for classification were not explicitly included in the eIMCI system but completion of assessment of these items was documented for both eIMCI and pIMCI.

**Table 1:** Completeness of assessment areas under paper-based and electronic systems for ten assessment areas programmed into eIMCI

**Assessment area from the IMCI protocol**	**System**				
	**Paper-based system**	**Electronic system**	**OR**^**†**^	**CI**	**p-value**
	**(N**_**p**_ **= 671)**	**(N**_**e**_ **= 550)**			
Vomiting everything	518 (77.2%)	526 (95.6%)	6.3	(1.2-34.5)	0.033
Ability to drink	464 (70.3%)	527 (95.8%)	9.5	(2.3-39.6)	0.002
Ability to breastfeed^‡‡^	310 (83.3%)	308 (97.8%)	7.8	(2.5-24.8)	<0.001
Convulsions^‡^	519 (77.7%)	544 (99.1%)	26.1	(9.9-68.7)	<0.001
Visibly awake^††^	540 (80.6%)	528 (96.2%)	5.6	(2.6-12.2)	<0.001
Diarrhea	581 (86.6%)	549 (99.8%)	92.1	(7.5-1118.8)	<0.001
Fever	656 (97.8%)	548 (99.6%)	6.6	(1.3-32.8)	0.022
Cough/difficulty breathing	602 (89.7%)	548 (99.6%)	27.4	(6.8-111.1)	<0.001
Ear problems	408 (60.8%)	545 (99.1%)	61.6	(18.4-206.4)	<0.001
Feeding change during illness^*^	377 (64.1%)	448 (92.4%)	6.8	(2.6-17.6)	<0.001

^†^ Population average from GEE analysis; ^‡^ N_p_ = 668, N_e_ = 549; ^††^ N_p_ = 670, N_e_ = 549; ^‡‡^ Limited to children under 24 months, N_p_ = 372, N_e_ = 315; ^*^ N_p_ = 588, N_e_ = 485.

**Table 2:** Completeness of assessment areas under paper-based and electronic systems for five assessment areas not programmed into eIMCI

**Assessment area from the IMCI protocol**	**System**				
	**Paper-based system**	**Electronic system**	**OR**^**†**^	**CI**	**p-value**
Child wasting^‡^	390 (58.5%)	297 (54.4%)	0.79	(0.53-1.2)	0.256
Palmer pallor^††^	369 (55.5%)	250 (48.9%)	0.61	(0.37-0.98)	0.042
Edema^‡‡^	65 (9.8%)	66 (12.2%)	1.5	(0.51-4.2)	0.482
Weight-for-height^*^	450 (67.2%)	381 (69.8%)	1.1	(0.39-3.0)	0.876
Vaccinations^*^	456 (68.1%)	391 (71.6%)	1.1	(0.43-3.0)	0.798

^†^ Population average from GEE analysis; ^‡^ N_p_ = 667, N_e_ = 546; ^**††**^ N_p_ = 665, N_e_ = 545; ^‡‡^ N_p_ = 664, N_e_ = 543; ^*^ N_p_ = 670, N_e_ = 546.

For secondary outcomes, we documented completed follow-up assessment questions (as appropriate) and agreement between the classification of the health worker and the IMCI expert on the four primary disease areas – pneumonia, dehydration (with diarrhea for less than 14 days), diarrhea (for more than 14 days) and malaria. For the comparison of diagnoses, the IMCI expert was used as the gold standard.

### Sample size and power

Based on the preliminary study, we anticipated 65% adherence to the assessment sections under pIMCI and 80% adherence under eIMCI. Assuming no clustering, 185 individuals were needed to achieve 90% power to detect a difference at a 0.05 significance level. During the design phase, we anticipated observing 30 patients per arm per clinic at 20 clinics, corresponding to an intraclass correlation (ICC) of 0.077. Because of logistical constraints, we only included 18 facilities in this study. However, power to detect this difference remained high at 87%.

### Statistical analysis

Basic demographics and disease profiles of the children seen under each system were compared using frequencies. The impact of the system on the completeness of assessment was assessed using generalized estimating equations with an exchangeable correlation matrix to account for the clustering by clinics. Because of the small number of clinics, all results were validated using the Wilcoxon signed-rank test. The Wilcoxon signed-rank test was also used to compare changes in the median number of follow-up questions (by symptom area) for each clinic. For each assessment area, the ICC was calculated by system. The percent agreement of classification between the clinical health worker and the IMCI expert was estimated with binomial confidence intervals for each disease area and tested for significant difference in percent agreement by system using generalized estimating equations. All analyses were completed in Stata/MP 11.0.

### Ethical issues

Consent for clinic participation was obtained from both the district health officer and the healthcare worker at the clinic. Consent was also obtained from the caregiver of each child observed. The study was approved by the institutional review boards for Harvard School of Public Health, Ifakara Health Institute and the National Institute of Medical Research in Tanzania.

## Results

We observed a total of 1221 visits – 671 paper, 550 electronic. The median number of observations per clinic under pIMCI was 33 (range:6–74) and under eIMCI was 28 (range: 15–80). Table 3 summarizes the characteristics of children observed by system. The demographics and disease profiles were similar between the two groups, with the only significant difference in the proportion of children with low weight.

**Table 3:** Characteristics of children observed under the two systems

	**Paper-based system**	**Electronic system**	
	**N = 671**	**N = 550**	p-value*
Female	49%	52%	0.295
Age Category			0.499
*less than 6 months*	11%	9%	
*6 months - 1 year*	18%	21%	
*1 year - 2 years*	26%	27%	
*2 years - 5 years*	44%	42%	
Median temperature, Celcius (IQR)	38 (37–39)	38 (37–39)	0.445
Presentation of illnesses according to expert classification			
Some pneumonia	23%	23%	0.665
Severe pneumonia	1%	2%	
Some dehydration	1%	1%	0.373
Severe dehydration	<1%	<1%	
Some diarrhea	<1%	<1%	1.000
Dysentery	2%	2%	
Some malaria	84%	82%	0.699
Severe malaria	2%	2%	
Low weight	3%	1%	0.047
Severely underweight	<1%	<1%	
Anemia	17%	14%	0.291
Severely anemic	<1%	<1%	
Any severe disease	8%	9%	0.535
Competing illnesses, pneumonia plus			
*Dehydration*	3%	1%	0.459
*Diarrhea*	1%	1%	1.000
*Malaria*	91%	89%	0.559
*Ear Infection*	1%	2%	0.336
*Low weight*	6%	2%	0.236
*Anemia*	20%	22%	0.670
Competing illnesses, malaria plus			
*Pneumonia*	26%	26%	0.832
*Dehydration*	2%	1%	0.244
*Diarrhea*	1%	2%	0.801
*Ear Infection*	2%	3%	0.272
*Low weight*	4%	1%	0.019
*Anemia*	18%	16%	0.458

* p-values comparing paper-based and electronic systems using Wilcoxon Rank-sum Test for temperature and Fisher’s Exact Test for remaining variables.

### Impact of electronic system on adherence to IMCI protocols

For the 15 critical assessment areas, ten were included on both eIMCI and pIMCI protocols (Table 1). For all ten areas, questions were more likely to be asked by the healthcare worker when using eIMCI compared to pIMCI (p < 0.05). Only 20.7% of children had all ten items assessed under the paper system compared to 70.9% under the electronic system (p < 0.001). For the five areas not included in the electronic protocols, the completeness of assessment was nearly identical under the paper and electronic systems (Table 2). For the full fifteen items, the proportion of complete assessments were low for both systems – 4.6% for pIMCI and 8.4% for eIMCI (p = 0.212).

Three symptom areas, namely cough/difficulty breathing, diarrhea, and fever, required additional follow-up questions to determine the severity of the final classification. For all sites and all symptoms, at least as many and often more questions were asked under the electronic system as compared to the paper system (p < 0.01 for all three assessment areas).

### Impact of electronic system on classification of diseases

For the four primary classification areas, the electronic protocols led to more accurate classification (Table 4). The difference was only significant for malaria. When looking at classification in all four areas, 82.7% of encounters had correct classification in all areas under pIMCI compared to 90.9% under eIMCI (p < 0.001). Table 4 also presents the difference in the classification of severe disease, which is distinguished from other categories of classification by the follow-up questions. However, the numbers of severe cases in all four disease areas are too small for a formal comparison.

**Table 4:** Accuracy of classification compared to IMCI expert

**Correct classification**	**System**			
	**Paper-based system**		**Electronic system**	
	**Percent of correct classification for all levels of disease (none, some, severe)**			
	**n/N**	**% (95% CI)**	**n/N**	**% (95% CI)**
Pneumonia	608/671	90.6%	513/550	93.3%
		(88.1%-92.7%)		(90.8%-95.2%)
Diarrhea	661/671	98.5%	549/550	99.8%
		(97.3%-99.3%)		(99.0%-100%)
Dehydration	663/671	98.8%	550/550	100%
		(97.7%-99.5%)		(99.3%-100%)
Malaria	627/671	93.4%	536/550	97.5%
		(91.3%-95.2%)		(95.8%-98.6%)
	**Percent of correct classification for severe disease**			
	**n/N**	**%**	**n/N**	**%**
Severe Pneumonia	2/9	22%	7/11	64%
Severe Diarrhea	10/15	67%	10/11	91%
Severe Dehydration	1/2	50%	2/2	100%
Severe Malaria	16/16	100%	10/11	91%

### Systems impact of electronic system

Table 5 presents the estimated ICC values by system and by assessment area. For eIMCI, of the ten areas included in the electronic protocol, seven had ICC values below 0.1. For the three areas with ICCs above 0.1, the ICC values were sensitive to one site performance. When the outlier site was removed from analysis for these three assessment areas under eIMCI, the ICC dropped below 0.1 (ICC = 0 for vomiting everything, ICC = 0.023 for ability to drink, ICC = 0.06 for feeding change during illness). For the corresponding ten assessment areas, the ICC was greater than 0.1 under pIMCI and remained above 0.1 for most of the corresponding assessment areas (7 out of 10) in the paper system even in the sensitivity analysis. For the five assessment areas not covered in the electronic system, the ICC for the paper and electronic system were universally above 0.1.

**Table 5:** Intraclass correlation, listed by specific assessment areas

	**System**			
	**Control (Paper-based system)**		**Electronic system**	
		**Included in the electronic protocol**		
Diarrhea	**0.33**	(0.15-0.50)	**0**	(0-0.02)
Vomiting	**0.39**	(0.17-0.53)	**0.69†**	(0.53-0.85)
Fever	**0.1†**	(0.02-0.18)	**0**	(0-0.02)
Cough	**0.28**	(0.12-0.45)	**0**	(0-0.02)
Ear problems	**0.48**	(0.28-0.67)	**0.01**	(0-0.04)
Ability to drink	**0.29**	(0.12-0.45)	**0.33†**	(0.15-0.50)
Convulsions	**0.45**	(0.25-0.64)	**0.01**	(0-0.05)
Visibly awake	**0.46**	(0.27-0.66)	**0.03**	(0-0.07)
Ability to breastfeed	**0.22†**	(0.07-0.38)	**0.07**	(0-0.16)
Ability to eat foods	**0.2‡**	(0.06-0.34)	**0.19†**	(0.06-0.32)
	**Not included in the electronic protocol**			
Child wasting	**0.78**	(0.64-0.91)	**0.91**	(0.85-0.97)
Palmer pallor	**0.54**	(0.35-0.74)	**0.72**	(0.57-0.88)
Edema	**0.42†**	(0.23-0.61)	**0.69**	(0.53-0.85)
Weight-for-height	**0.36**	(0.18-0.55)	**0.43**	(0.24-0.61)
Vaccinations	**0.35**	(0.17-0.53)	**0.41**	(0.22-0.60)

ICC - Intraclass correlation; † ICC sensitive to one site performance and drops below 0.1 if site removed; ‡ ICC sensitive to two sites and drops below 0.1 if either of these sites is removed.

The average time per visit under the current paper-based system was 8.98 minutes (standard error, 0.20). For eIMCI, the average visit length was 9.06 minutes (standard error, 0.20). The length of visit was not significantly different between the two systems (p = 0.996). Trainers noted the time required to train health workers in use of the electronic protocols, and consistent with previous studies, the time required for training was 1–2 hours (not including basic IMCI training that all health workers had undergone previously through the Ministry of Health) [14].

## Discussion

The results of this study provide strong evidence supporting the potential of mobile health to improve quality of health care in low-income countries, including thoroughness of assessment and accuracy of classification. For diagnoses, the agreement between the health worker and IMCI expert was higher under eIMCI compared to pIMCI, but was only significantly different for one of the four classification areas. An interesting comparison between pIMCI and eIMCI is the impact on classification of severe disease, which is distinguished from non-severe disease through the completion of the follow-up assessment questions and is critical for identification because these are the leading causes of preventable death in children [17]. Showing significance with this data is impossible because of the few cases of severe disease. Therefore the true impact of mobile technology on the classification of severe disease and impact on health outcomes of children must be evaluated in future studies.

Another improvement observed under eIMCI was better consistency in care. The ICC values under pIMCI were very high, suggesting that the completeness of assessment very much depends on the individual conducting the assessment. In comparison, the assessment areas included in eIMCI had much lower values of ICC. These results, combined with the proportion of visits with complete assessments, imply that assessments are not only better but are also more consistent across clinics with the support of mobile technology.

The mechanisms for the improvements observed in this study are supported by results from a qualitative study conducted at these sites [18]. Health workers indicated a stronger preference for the PDA compared to the paper charts used to support pIMCI implementation, both because the tool was easier to use and because of the perceived improvement in confidence from the children and caregivers. Indeed, the caregivers also expressed a preference because they believed the health workers were better equipped with the PDA and because they believed they were more engaged when eIMCI was used.

There are several limitations to this study. First, the number of clinics included in the study were fewer than originally planned and the ICC values for pIMCI were larger than that used in the design phase. Fortunately, the effect of eIMCI was greater than anticipated and so we were able to achieve statistical significance. These estimated ICC values will be used to improve study designs in the future. Another limitation in the design was the before-after nature of the assessments as such designs have the potential for temporal confounding. Given the logistical challenges and early stages of evaluation for eIMCI, a cluster randomized trial was not feasible for this study. However, in this case, we believe the introduction of temporal confounding is minimal since both phases of data collection at a site occurred within three weeks. A third limitation is the possible Hawthorne effect, since there was an observer present in the room during the IMCI assessment. While such effect is inevitable when using observers, we feel this effect would only dilute the estimated impact of eIMCI. A further limitation is that the study focused exclusively on assessment and classification elements. Correct treatment and counseling are critical components of the IMCI encounter, and to fully understand the usefulness of eIMCI, these elements must be included in future studies. Finally, an additional limitation is that five of the 15 critical assessment areas were not included in the eIMCI protocol. The elements omitted were not critical for the classification in the four main disease areas, but have been noted as important and will be included in future iterations. Further, the omission of these items provided an important insight. For items programmed into the system, the observers documented improvement, for the others no improvement was observed. This suggests that any desirability bias was minimal.

Although this study looked narrowly at adherence to IMCI protocols and subsequent classification, there are many implications of its results. Perhaps most significant is the role that computerized protocols can have on effective task shifting in low-income countries. Given the severe shortages of trained health personnel in low-income countries [19], there is an urgent need to find ways to engage lesser-trained health personnel to accurately diagnose and treat patients while maintaining high quality of care. While electronic technology cannot replace the role of training and supervision, such tools can complement these existing mechanisms to ensure consistency and quality. A second important implication of this study is the ability to build other components onto an electronic decision support system including a medical record system. The eIMCI system that is currently being developed facilitates the transfer of data about each patient and each encounter to a centrally stored patient record. This record would enable the health worker to track a child’s weight, immunization status or other health problems over time. This improves the ability of the IMCI protocols to accurately assess a child’s condition, and there is evidence from other applications that an effective electronic medical record has the ability to improve care [20]. Further, this data can also be made available for disease surveillance, remote supervision and routine reporting improving information available to health planners and managers on a timely basis. A third benefit of the approach used in this study is the ability to quickly change protocols as policies change [21]. This has been a problem faced by IMCI since a change in protocols for the treatment of fever has required a complete retraining and reprinting of all booklets to include the newer drugs and incorporate the use of rapid diagnostic tests. We are currently engaged in studies to better understand the impact of mobile technology, and specifically eIMCI, on these areas of task-shifting, contributing to a functional electronic medical record, and improving the ability to update protocols in a timely manner. Further, we are presently studying the sustained impact of eIMCI on adherence and classification and the impact of the protocol on health outcomes after 12 months of implementation.

## Conclusions

The emerging field of mobile and electronic health is showing great promise in improving both the delivery and monitoring of health care in low-income countries. This study demonstrates its benefit to deliver a proven standard of care: IMCI in a rural and semi-urban area of Tanzania. While this study showed improved adherence to a specific set of clinical guidelines (IMCI) it seems likely that this same result could be achieved with the use of electronic guidelines in other areas such as maternal health, chronic illness and infectious disease where national guidelines are often available but used sporadically. Based on this finding, the authors anticipate a much broader use of mobile technology for the delivery of clinical standards aimed at improving clinical care in low-income countries, including studies investigating the benefits with other clinical protocols and with other cadres of healthcare workers.

## Competing interests

MM, BHG, MN, and NL either were or currently are employees of D-Tree International. D-Tree International is non-profit organization that developed the eIMCI system under evaluation. Data collection was led by an outside party, Ifakara Health Institute.

## Authors’ contributions

All authors were involved in the design, implementation and analysis of the study. MM was the PI of the project and led the writing of the manuscript. BHG led the statistical analysis and assisted with the writing of the manuscript. DM led the data collection team. MN assisted with data collection and data input. NL led the software team. All authors read and approved the final manuscript.

## Pre-publication history

The pre-publication history for this paper can be accessed here:

http://www.biomedcentral.com/1472-6947/13/95/prepub
